# Epigenome-wide association study identifies novel genes associated with ischemic stroke

**DOI:** 10.1186/s13148-023-01520-x

**Published:** 2023-06-27

**Authors:** Hao Peng, Helena Palma-Gudiel, Carolina Soriano-Tarraga, Jordi Jimenez-Conde, Mingzhi Zhang, Yonghong Zhang, Jinying Zhao

**Affiliations:** 1 Department of Epidemiology, School of Public Health and Jiangsu Key Laboratory of Preventive and Translational Medicine for Geriatric Diseases, Medical College of Soochow University, 199 Renai Road, Suzhou, 215123 China; 2 Department of Epidemiology, College of Public Health and Health Professions and College of Medicine, University of Florida, 2004 Mowry Road, Gainesville, FL 32610 USA; 3 Neurovascular Research Group, Department of Neurology of Hospital del Mar-IMIM (Institut Hospital del Mar d’Investigacions Mèdiques), Universitat Autònoma de Barcelona/DCEXS, Universitat Pompeu Fabra, Barcelona, Spain; 4 Department of Psychiatry, Washington University School of Medicine, St. Louis, MO USA; 5 Hope Center for Neurological Disorders, Washington University School of Medicine, St. Louis, MO USA; 6 NeuroGenomics and Informatics, Department of Psychiatry, Washington University in St. Louis, St. Louis, USA

**Keywords:** DNA methylation, Ischemic stroke, EWAS, CRISPR/dCas9-Dnmt3a, Chinese population

## Abstract

**Background:**

DNA methylation has previously been associated with ischemic stroke, but the specific genes and their functional roles in ischemic stroke remain to be determined. Here we aimed to identify differentially methylated genes that play a functional role in ischemic stroke in a Chinese population.

**Results:**

Genome-wide DNA methylation assessed with the Illumina Methylation EPIC Array in a discovery sample including 80 Chinese adults (40 cases *vs*. 40 controls) found that patients with ischemic stroke were characterized by increased DNA methylation at six CpG loci (individually located at *TRIM6*, *FLRT2*, *SOX1*, *SOX17*, *AGBL4,* and *FAM84A*, respectively) and decreased DNA methylation at one additional locus (located at *TLN2*). Targeted bisulfite sequencing confirmed six of these differentially methylated probes in an independent Chinese population (853 cases *vs*. 918 controls), and one probe (located at *TRIM6*) was further verified in an external European cohort (207 cases *vs*. 83 controls). Experimental manipulation of DNA methylation in engineered human umbilical vein endothelial cells indicated that the identified differentially methylated probes located at *TRIM6*, *TLN2,* and *FLRT2* genes may play a role in endothelial cell adhesion and atherosclerosis.

**Conclusions:**

Altered DNA methylation of the *TRIM6*, *TLN2,* and *FLRT2* genes may play a functional role in ischemic stroke in Chinese populations.

**Supplementary Information:**

The online version contains supplementary material available at 10.1186/s13148-023-01520-x.

## Background

Stroke is one of the leading causes of mortality and morbidity worldwide, second only to ischemic heart disease (IHD) [1]. In China, stroke mortality surpasses that of IHD, accounting for around one-third of all stroke cases worldwide [2]. Ischemic stroke (IS) constitutes more than 80% of all stroke cases [3]. The etiology of IS is multifactorial, involving both genetic and environmental factors [4, 5]. Despite the known contribution of genetic factors to the etiology of stroke, genetic variants identified so far explain less than 10% of the disease variability, suggesting that other factors may also play an important role in IS. Additionally, identified genetic risk variants for stroke confer race-specific effects [6], revealing the existence of underlying biological mechanisms that might explain the variability of stroke prevalence across different ancestries. The epigenome responds to a wide range of environmental stressors, providing a mechanism through which environmental factors affect the susceptibility to human complex diseases, including IS. Several epigenome-wide association studies (EWAS) have reported associations of altered DNA methylation with IS [7–[12]. However, prior studies were mostly conducted in European populations and results were largely inconsistent. Moreover, few studies have attempted to functionally validate the identified epigenetic markers in cellular models [13]. Here, we used a multi-stage design to identify DNA methylation alterations associated with IS in Chinese participants: (1) EWAS in a discovery Chinese cohort (discovery stage, n = 80); (2) confirmation and fine mapping of candidate regions by targeted methylation sequencing in an independent Chinese population (internal validation, n = 1,771); (3) replication in a European population (external replication, n = 390); and (4) functional validation of top hits in an endothelial cell model via the CRISPR/dCas9-Dnmt3a system.

## Results

**Fig. 1:**
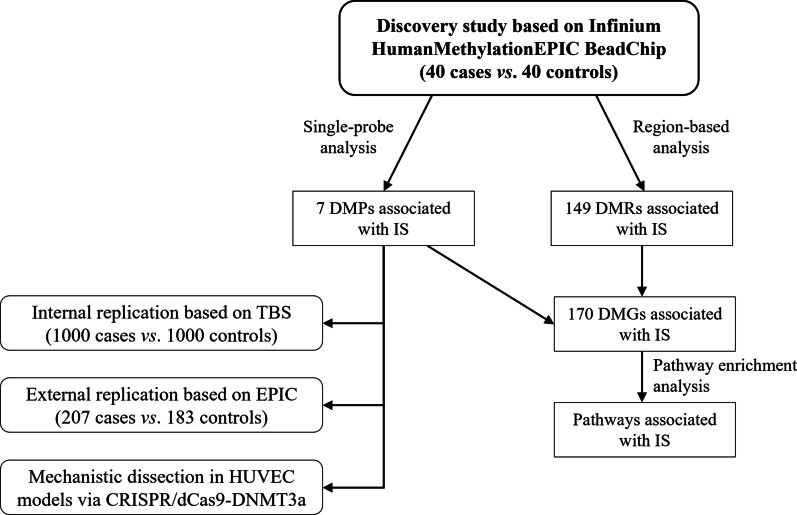
A flowchart illustrating the study design and selection of participants. In the discovery stage, 40 CATIS IS cases and 40 PMMS controls were randomly selected to identify potential epigenetic markers of IS. From the remaining CATIS and PMMS participants, 1000 cases and 1000 age- and sex-matched controls were randomly selected as an internal replication sample to further confirm epigenetic markers of IS; after QC, 853 cases and 918 controls remained for downstream analysis. Additionally, an external cohort of 390 Spanish participants was used for further validation of the DMPs identified. To test the causal effect on gene expression of DNA methylation at loci identified in the aforementioned stages, human umbilical vein endothelial cell (HUVEC) models were epigenetically modified via CRISPR/dCas9-Dnmt3a. DMG, differentially methylated gene; DMP, differentially methylated probe; DMR, differentially methylated region; HUVEC, human umbilical vein endothelial cell; IS, ischemic stroke; TBS, targeted bisulfite sequencing

The study design is illustrated in Fig. 1. Genome-wide DNA methylation was assessed with the Illumina Methylation EPIC Array in a discovery sample including 80 Chinese adults (40 cases *vs*. 40 controls). Differentially methylated probes (DMPs) identified were further confirmed by targeted bisulfite sequencing (TBS) in an independent sample (n = 1,771), followed by external replication in a European cohort (n = 390). The potential functional role of DMPs was verified in a CRISPR/dCas9-Dnmt3a model in human umbilical vein endothelial cells (HUVECs). Clinical characteristics of participants in each stage are shown in Table 1. Compared with healthy controls, IS cases had more risk factors such as hypertension, diabetes, and dyslipidemia in participants at all stages.

**Table 1:** Clinical characteristics of study participants in the discovery and replication samples at enrollment

	Discovery cohort (n = 80)			Internal validation (n = 1,771)			External replication (n = 390)		
	Controls N = 40	Cases N = 40	*P*-value	Controls N = 918	Cases N = 853	*P*-value	Controls N = 183	Cases N = 207	*P*-values
Age, years	65.6 ± 6.2	65.6 ± 6.2	0.986	61.2 ± 12.2	62.5 ± 12.1	0.026	64	71	< 0.001
Sex, men (%)	30 (75)	30 (75)	1.000	503 (55)	453 (53)	0.507	(48)	(64)	< 0.001
Current smoking, n (%)	16 (40)	14 (35)	0.817	348 (38)	294 (34)	0.118	2 (1)	49 (22)	< 0.001
Current drinking, n (%)	24 (60)	15 (37.5)	0.074	253 (28)	222 (26)	0.500	NA	NA	NA
Hypertension	5 (12.5)	27 (67.5)	< 0.001	325 (35)	670 (79)	< 0.001	107 (59)	74 (34)	< 0.001
BMI, kg/m^2^	24.7 ± 3.1	24.8 ± 3.0	0.825	22.4 ± 3.4	25.1 ± 3.4	< 0.001	28.1	26.6	< 0.001
SBP, mmHg	129.6 ± 9.1	165.7 ± 18.1	< 0.001	134.6 ± 20.7	168.1 ± 16.8	< 0.001	NA	NA	NA
DBP, mmHg	77.9 ± 6.8	94.7 ± 10.9	< 0.001	80.0 ± 10.8	97.0 ± 10.7	< 0.001	NA	NA	NA
Fasting glucose, mmol/L, diabetes mellitus*, n (%)	5.5 ± 1.1	7.2 ± 3.0	0.001	5.0 ± 1.2	6.8 ± 2.8	< 0.001	30 (16)	81 (37)	< 0.001
Triglycerides, mmol/L Hyperlipidemia**†**, n (%)	1.3 ± 0.7	1.6 ± 1.1	0.244	1.6 ± 1.2	1.9 ± 5.0	0.054	124 (68)	123 (56)	< 0.001
Total cholesterol, mmol/L	5.3 ± 0.9	5.0 ± 1.3	0.402	4.7 ± 1.0	5.1 ± 1.2	< 0.001	NA	NA	NA
LDL cholesterol, mmol/L	3.1 ± 0.8	2.8 ± 1.2	0.216	3.2 ± 1.0	2.9 ± 1.0	< 0.001	NA	NA	NA
HDL cholesterol, mmol/L	1.5 ± 0.4	1.4 ± 0.4	0.168	1.3 ± 0.3	1.3 ± 0.4	0.032	NA	NA	NA

All results are expressed as mean ± SD, unless otherwise noted. *Fasting glucose was not available in the external replication cohort, and thus presence/absence of diabetes mellitus was used for comparison. †Total triglycerides and cholesterol were not available in the external replication cohort; thus, presence/absence of hyperlipidemia was used for comparison. BMI, body mass index; DBP, diastolic blood pressure; IS, ischemic stroke; SBP, systolic blood pressure

### DMPs identified in association with IS

**Fig. 2:**
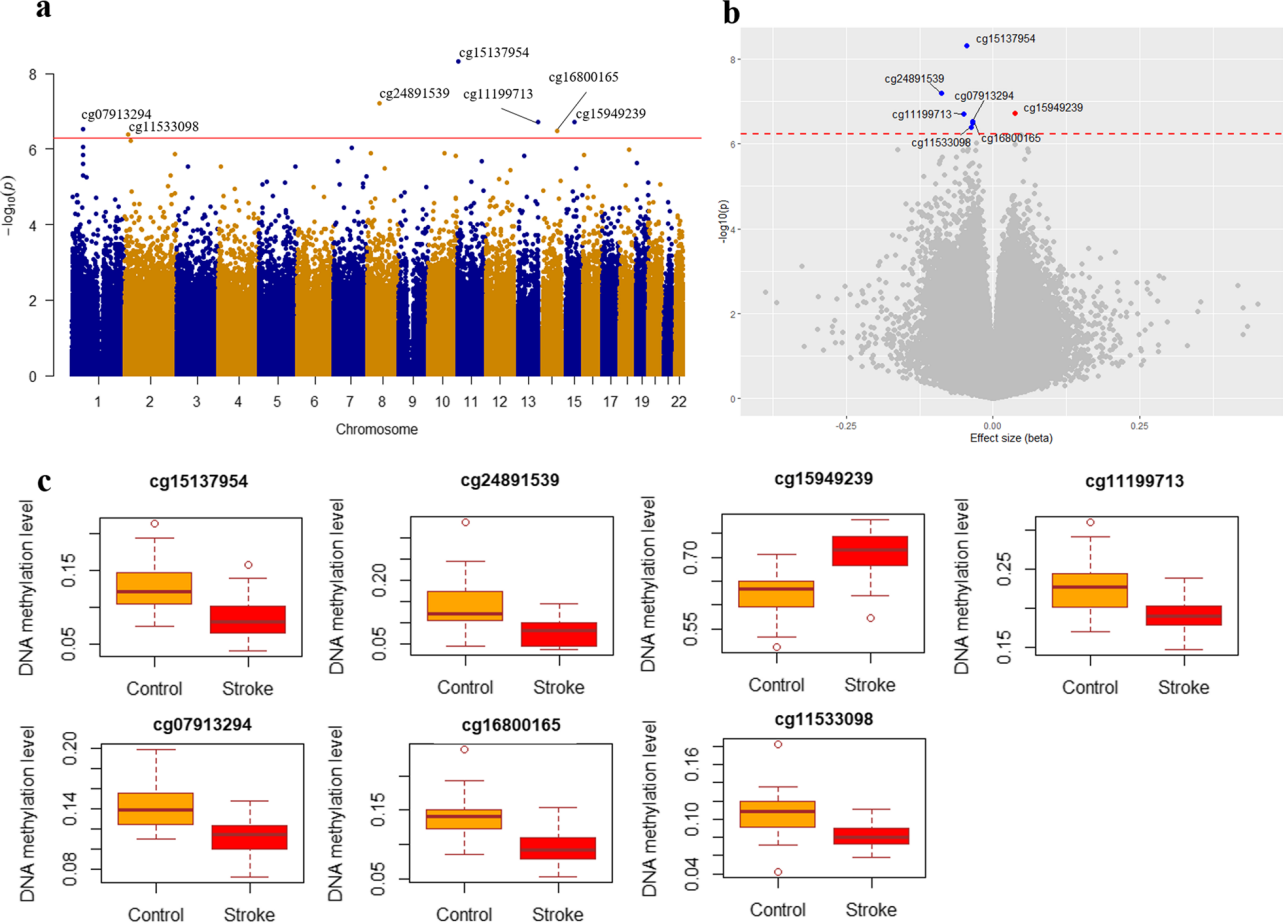
EWAS in the discovery stage. Manhattan (**a**) and volcano (**b**) plots displaying the associations of DNA methylation at 815,017 CpGs with IS in a Chinese sample (40 cases and 40 matched controls). In the Manhattan plot, X-axis indicates the chromosome position; Y-axis indicates the –log_10_ *P*-values. The red line denotes the significance threshold at q-value < 0.05. In the volcano plot, blue and red dots highlight significantly hypomethylated and hypermethylated CpG sites, respectively, in cases with respect to controls. **c** Box plots showing the differences in mean DNA methylation level between cases and controls at the 7 CpG sites significantly associated with IS in the discovery stage

Of the 815,017 CpG sites tested, single-probe analysis identified 46 differentially methylated positions (DMPs) associated with IS at *P* < 1.0 × 10^–5^ (Table 2), after adjusting for potential confounding factors in the discovery sample. Seven (out of 46) DMPs had q < 0.05 (Fig. 2) after further correction for multiple testing via FDR. Of these 7 DMPs, 6 DMPs (cg15137954 in *TRIM6*, cg11199713 in *SOX1*, cg07913294 in *AGBL4*, cg16800165 in *FLRT2*, cg11533098 in *FAM84A*, and cg15949239 in *TLN2*) were successfully confirmed (q ≤ 0.05) by targeted bisulfite sequencing (TBS) in an independent Chinese population comprising 853 cases and 918 controls (Table 3). In addition, altered DNA methylation at neighboring CpGs showed significant associations with IS in the same directions (region-based *P* < 5 × 10^–7^). Among these 6 DMPs validated in the Chinese cohorts, one probe (cg15137954) was also significantly associated with IS (same direction) in the European cohort (multiple testing adjusted *P* = 0.01). Complete statistics for the external validation are included in Table 3.

**Table 2:** Significant DMPs associated with ischemic stroke in the discovery sample (P < 1 × 10^–5^)

CpG	Genomic coordinates	Nearest gene	Gene location	Effect size*	P-value	q-value**†**
cg15137954	Chr11:5618023	*TRIM6*	TSS200	− 0.04	4.76 × 10^–9^	0.004
cg24891539	Chr8:55370407	*SOX17*	TSS200	− 0.09	6.28 × 10^–8^	0.025
cg15949239	Chr15:62988566	*TLN2*	Body	0.04	1.93 × 10^–7^	0.039
cg11199713	Chr13:112721773	*SOX1*	TSS200	− 0.05	1.94 × 10^–7^	0.039
cg07913294	Chr1:50489574	*AGBL4*	5'UTR	− 0.04	2.98 × 10^–7^	0.044
cg16800165	Chr14:85996364	*FLRT2*	TSS200	− 0.04	3.24 × 10^–7^	0.044
cg11533098	Chr2:14775132	*FAM84A*	3'UTR	− 0.04	4.09 × 10^–7^	0.048
cg12076203	chr2:27145848	*DPYSL5*	Body	0.05	6.00 × 10^–7^	0.061
cg20931033	chr1:50489577	*AGBL4*	1stExon	− 0.05	8.60 × 10^–7^	0.070
cg17814180	chr7:86274020	*GRM3*	1stExon	− 0.03	9.49 × 10^–7^	0.070
cg16586689	chr18:46082474	*CTIF*	5'UTR	0.04	1.02 × 10^–6^	0.070
cg27646412	chr10:77501817	*C10orf11*	TSS1500	0.06	1.31 × 10^–6^	0.070
cg15852516	chr8:17270812	*MTMR7*	1stExon	− 0.04	1.31 × 10^–6^	0.070
cg21426003	chr2:237076811	*GBX2*	TSS200	− 0.16	1.38 × 10^–6^	0.070
cg08582701	chr16:6070005	*A2BP1*	5'UTR	− 0.05	1.40 × 10^–6^	0.070
cg14615336	chr1:50489567	*AGBL4*	1stExon	− 0.03	1.43 × 10^–6^	0.070
cg13929328	chr10:129535898	*FOXI2*	1stExon	− 0.04	1.50 × 10^–6^	0.070
cg03135713	chr13:46961897	*C13orf18*	TSS1500	0.06	1.54 × 10^–6^	0.070
cg23093589	chr7:20824932	*SP8*	Body	− 0.06	2.07 × 10^–6^	0.085
cg12789173	chr11:118084192	*AMICA1*	TSS200	− 0.09	2.08 × 10^–6^	0.085
cg06950070	chr19:6502301	*TUBB4*	1stExon	0.05	2.33 × 10^–6^	0.091
cg05387519	chr1:50489730	*AGBL4*	TSS200	− 0.09	2.51 × 10^–6^	0.093
cg16532100	chr5:171611704	*STK10*	Body	− 0.03	2.83 × 10^–6^	0.095
cg01756381	chr4:11430693	*HS3ST1*	TSS200	− 0.09	2.89 × 10^–6^	0.095
cg05362127	chr3:55515543	*WNT5A*	TSS200	− 0.04	2.92 × 10^–6^	0.095
cg02294868	chr8:79579003	*ZC2HC1A*	5'UTR	− 0.02	3.29 × 10^–6^	0.100
cg18517195	chr15:71184476	*LRRC49;THAP10*	TSS1500;1stExon	− 0.04	3.32 × 10^–6^	0.100
cg26026615	chr12:115130855			− 0.06	3.67 × 10^–6^	0.107
cg00787188	chr2:213403315	*ERBB4*	1stExon	− 0.05	4.93 × 10^–6^	0.134
cg13424673	chr1:50489744	*AGBL4*	TSS200	− 0.03	4.93 × 10^–6^	0.134
cg00903099	chr7:154862441	*HTR5A*	TSS200	− 0.08	5.29 × 10^–6^	0.139
cg24637364	chr1:66259084	*PDE4B*	5'UTR	− 0.12	5.74 × 10^–6^	0.146
cg10862431	chr12:63544783	*AVPR1A*	TSS200	− 0.04	6.24 × 10^–6^	0.154
cg00107187	chr14:105070998	*TMEM179*	1stExon	− 0.05	6.90 × 10^–6^	0.164
cg21590264	chr5:37834850	*GDNF*	1stExon	− 0.04	7.39 × 10^–6^	0.164
cg21180443	chr11:66984956	*KDM2A*	TSS200	− 0.02	7.51 × 10^–6^	0.164
cg03609960	chr12:99288805	*ANKS1B*	TSS200	− 0.04	7.61 × 10^–6^	0.164
cg01546873	chr5:122523277	*PRDM6*	3'UTR	0.05	7.78 × 10^–6^	0.164
cg08146323	chr19:57183118	*ZNF835*	1stExon	− 0.07	7.88 × 10^–6^	0.164
cg07664198	chr7:136553882	*CHRM2*	1stExon	− 0.04	8.07 × 10^–6^	0.164
cg12842973	chr20:56650428			− 0.06	8.54 × 10^–6^	0.166
cg00455526	chr7:28996196	*TRIL*	1stExon	− 0.10	8.67 × 10^–6^	0.166
cg19524676	chr5:17218547	*BASP1;* *LOC285696*	5'UTR; TSS1500	− 0.04	8.74 × 10^–6^	0.166
cg15439862	chr18:28622593	*DSC3*	1stExon	− 0.04	9.05 × 10^–6^	0.168
cg05824976	chr2:203737195	*ICA1L*	TSS1500	0.07	9.60 × 10^–6^	0.172
cg07514158	chr16:24267526	*CACNG3*	1stExon	− 0.03	9.68 × 10^–6^	0.172

^*^Difference in mean DNA methylation level between cases and controls (i.e., negative values indicate stroke cases were hypomethylated; positive values indicate stroke cases were hypermethylated at the specific CpG site)

^†^Adjusting for a total of 815,017 CpG sites using the false discovery rate (FDR) method

**Table 3:** DMPs significantly associated with ischemic stroke in the discovery (q < 0.05) and replication samples

CpG	Genomic coordinates	Nearest gene	Gene location	Discovery sample (n = 80)			Internal replication (n = 1,771)			External replication (n = 390)		
				Δ methylation*	P-value	q-value†	Δ methylation*	P-value	q-value^c^	Δ methylation*	P-value	q-value‡
cg15137954	Chr11:5618023	*TRIM6*	TSS200	− 4.4%	4.76 × 10^−09^	0.004	− 2.1%	1.28 × 10^−41^	7.65 × 10^−41^	− 4.9%	0.01	0.05
cg24891539	Chr8:55370407	*SOX17*	TSS200	− 6.5%	6.28 × 10^−08^	0.025	NA	NA	NA	0.5%	0.04	0.08
cg15949239	Chr15:62988566	*TLN2*	Gene body	8.5%	1.93 × 10^−07^	0.039	1.9%	0.053	0.053	0.2%	0.62	0.62
cg11199713	Chr13:112721773	*SOX1*	TSS200	− 3.4%	1.94 × 10^−07^	0.039	− 1.2%	2.27 × 10^−08^	2.73 × 10^−08^	0.4%	0.31	0.44
cg07913294	Chr1:50489574	*AGBL4*	5'UTR	− 2.9%	2.98 × 10^−07^	0.044	− 1.1%	1.34 × 10^−09^	2.01 × 10^−09^	0.6%	0.09	0.15
cg16800165	Chr14:85996364	*FLRT2*	TSS200	− 4.5%	3.24 × 10^−07^	0.044	− 4.4%	1.25 × 10^−09^	2.01 × 10^−09^	0.2%	0.50	0.59
cg11533098	Chr2:14775132	*FAM84A*	3'UTR	− 2.6%	4.09 × 10^−07^	0.048	− 0.6%	1.33 × 10^−13^	4.00 × 10^−13^	0.7%	0.01	0.05

^*^Difference in mean DNA methylation level between cases and controls. †Adjusting for a total of 815,017 CpG sites using the false discovery rate (FDR) method. ‡Multiple testing was corrected by FDR. One probe (cg24891539) failed to be confirmed by targeted bisulfite sequencing in the internal validation cohort

### Functional validation in cellular models

**Fig. 3:**
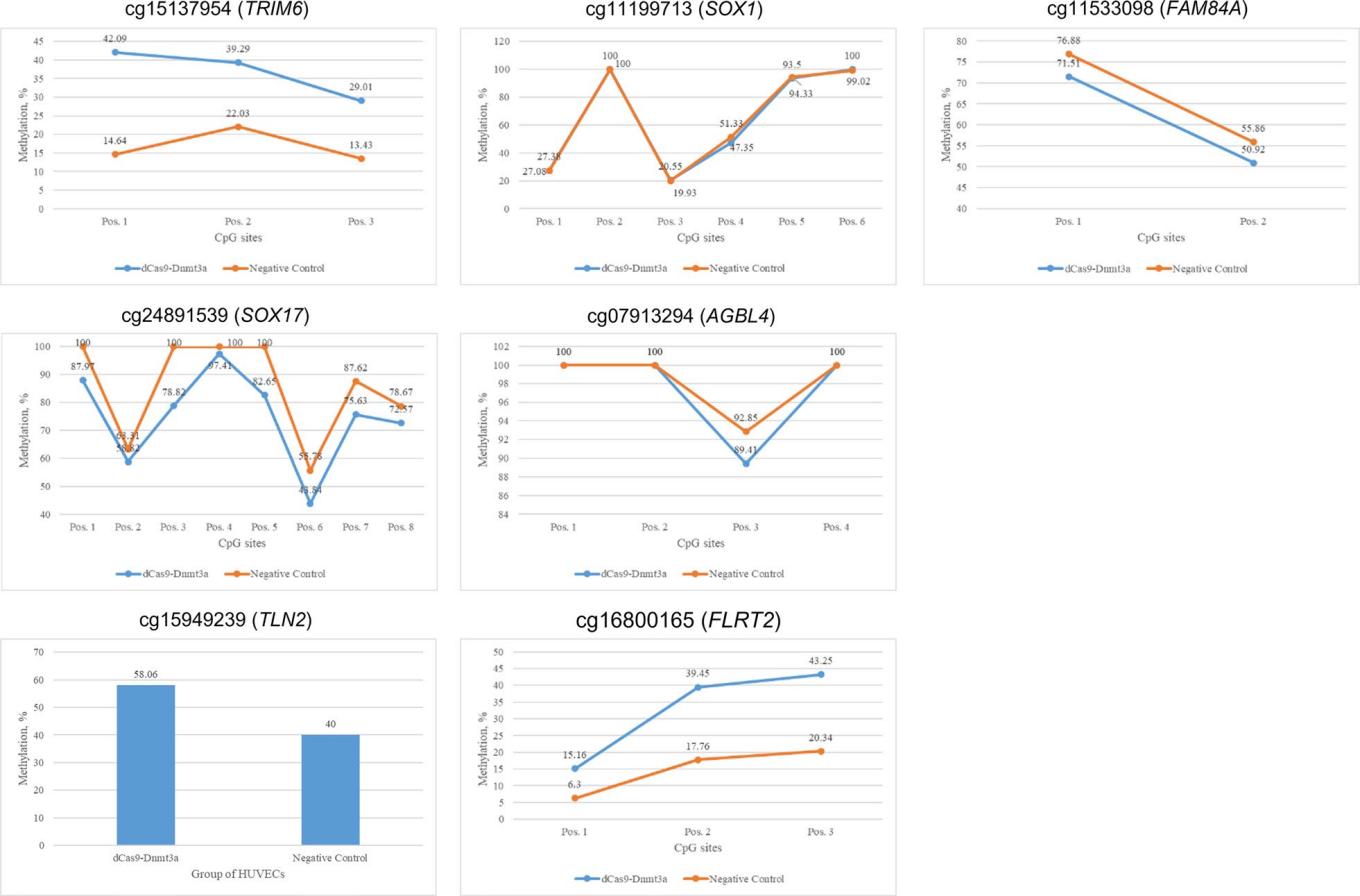
Validation of the CRISPR/dCas9-Dnmt3a model construction in human umbilical vein endothelial cells (HUVEC). Pyrosequencing was used to measure DNA methylation levels in the engineered and control cells. Successful model construction was considered when DNA methylation level of the engineered cells was higher than that of the negative control (i.e., *TRIM6*, *FLRT2*, and *TLN2* genes). No significant differences in DNA methylation levels were observed for *SOX1*, *FAM84A*, *SOX17*, and *AGBL4* genes

**Fig. 4:**
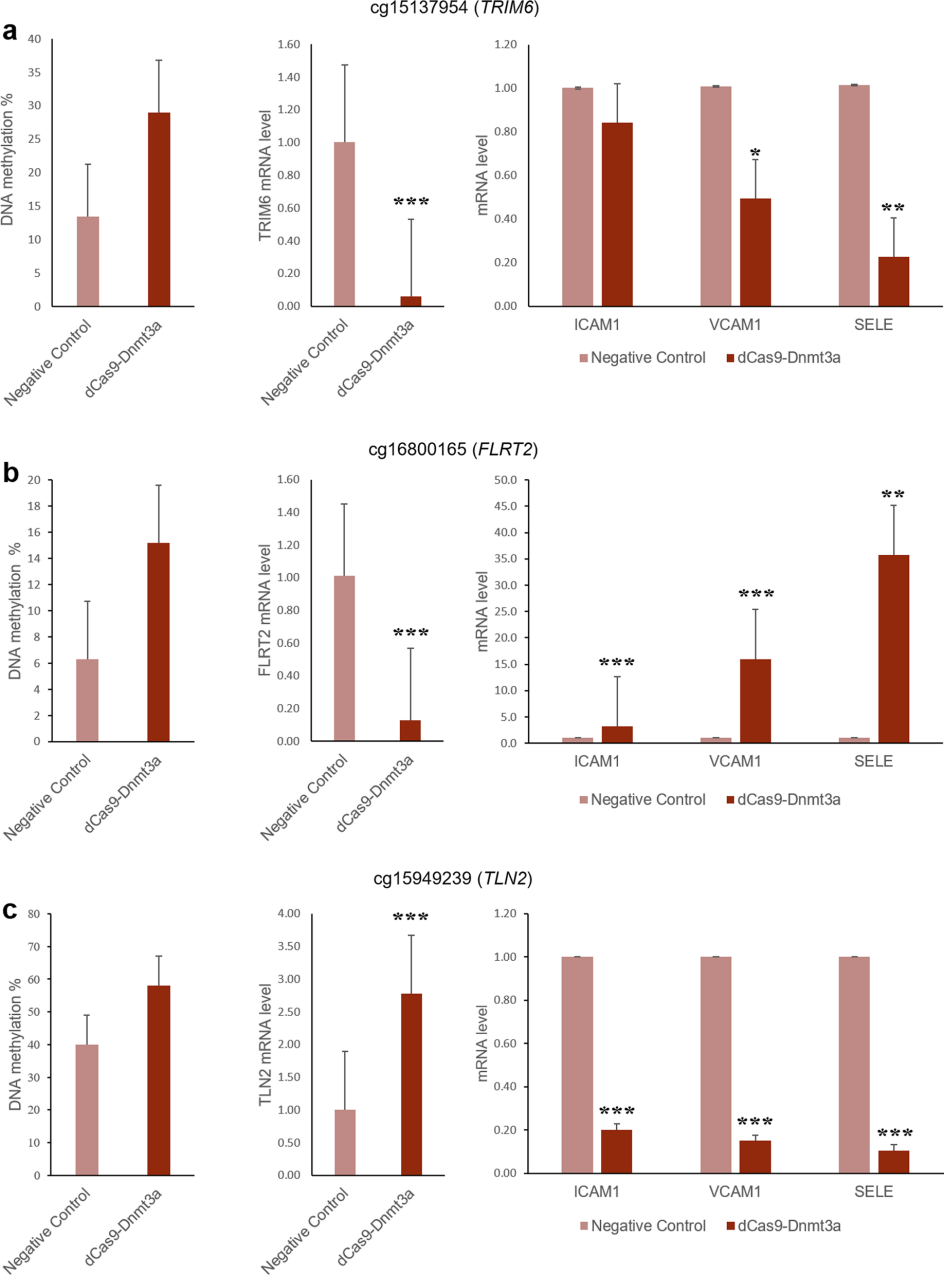
The expression of differentially methylated genes and genes encoding endothelial cell adhesion markers (i.e., *ICAM1*, *VCAM1*, and *SELE*) in site-specific methylation model HUVECs (dCase9-Dnmt3a) and negative control HUVECs. (**a**) *TRIM6* model: (left) methylation level at cg15137954 is higher in dCas9-Dnmt3a engineered cells than in negative control cells; (center) higher methylation is associated with decreased TRIM6 expression; (right) decreased expression of VCAM1 and SELE can be observed in dCas9-Dnmt3a engineered cells. (**b**) *FLRT2* model: (left) methylation level at cg16800165 is higher in dCas9-Dnmt3a engineered cells than in negative control cells; (center) *FLRT2* expression is significantly decreased in dCas9-Dnmt3a engineered cells; (right) expression of genes encoding endothelial cell adhesion markers is significantly increased in dCas9-Dnmt3a cells. (**c**) *TLN2* model: (left) methylation at cg15949239 is higher in dCas9-Dnmt3a cells than in the negative control cells; (center) *TLN2* expression is significantly increased in dCas9-Dnmt3a cells; (right) expression of genes encoding endothelial cell adhesion markers is significantly decreased in dCas9-Dnmt3a engineered cells when compared to negative control cells. Relative mRNA expression of dCase9-Dnmt3a to negative control was calculated using the 2(-Delta Delta C(T)) method and normalized with the reference gene *Actin* (3 repeated measurements per group). Data are mean ± SEM (standard error of mean). A Student t test was used to compare the group difference in gene expression levels. *P < 0.05; **P < 0.01; ***P < 0.001

To test the potential impact of DNA methylation at the CpG sites identified in a relevant tissue for stroke pathogenesis, the CRISPR/dCas9-Dnmt3a system was used to experimentally induce increased DNA methylation levels in the gene regions of interest in human umbilical endothelial cells (HUVECs). After generating the epigenetically modified cells, pyrosequencing was used to check the validity of the model, i.e., to ascertain increased DNA methylation levels in the CRISPR-modified cells. Of the 7 DMPs identified in the discovery stage, DNA methylation levels of 3 CpG sites (cg15137954, cg16800165, and cg15949239) in the engineered HUVECs were two times higher when compared to the negative control cells, denoting successful construction of the cellular models by the CRISPR/dCas9-Dnmt3a system (Fig. 3). The other 4 CpG sites (cg24891539, cg11199713, cg07913294, and cg11533098) exhibited similar methylation levels when comparing the two groups (Fig. 3). Gene expression analysis by qRT-PCR revealed that, compared to the negative control cells, the dCas9-Dnmt3a engineered HUVECs with an increased methylation level at cg15137954 (located in the *TRIM6* gene) resulted in decreased expression of the *TRIM6, VCAM1,* and *SELE* genes (Fig. 4a). In contrast, enhanced methylation at cg16800165 (located in *FLRT2*) led to reduced expression of the *FLRT2* gene and increased expression of the endothelial cell adhesion marker genes *ICAM1*, *VCAM1*, and *SELE* (Fig. 4b). In addition, increased DNA methylation at cg15949239 (located at *TLN2*) was associated with a heightened expression of the *TLN2* gene together with decreased expression of the cell adhesion marker genes *ICAM1*, *VCAM1*, and *SELE* (Fig. 4c). Together, these findings suggest that DNA methylation levels at the identified CpG sites regulate gene expression of their corresponding genes; additionally, it points to a functional role of the identified DMPs in endothelial cell activation and vascular inflammation.

### DMRs identified in association with IS in the discovery sample

**Fig. 5:**
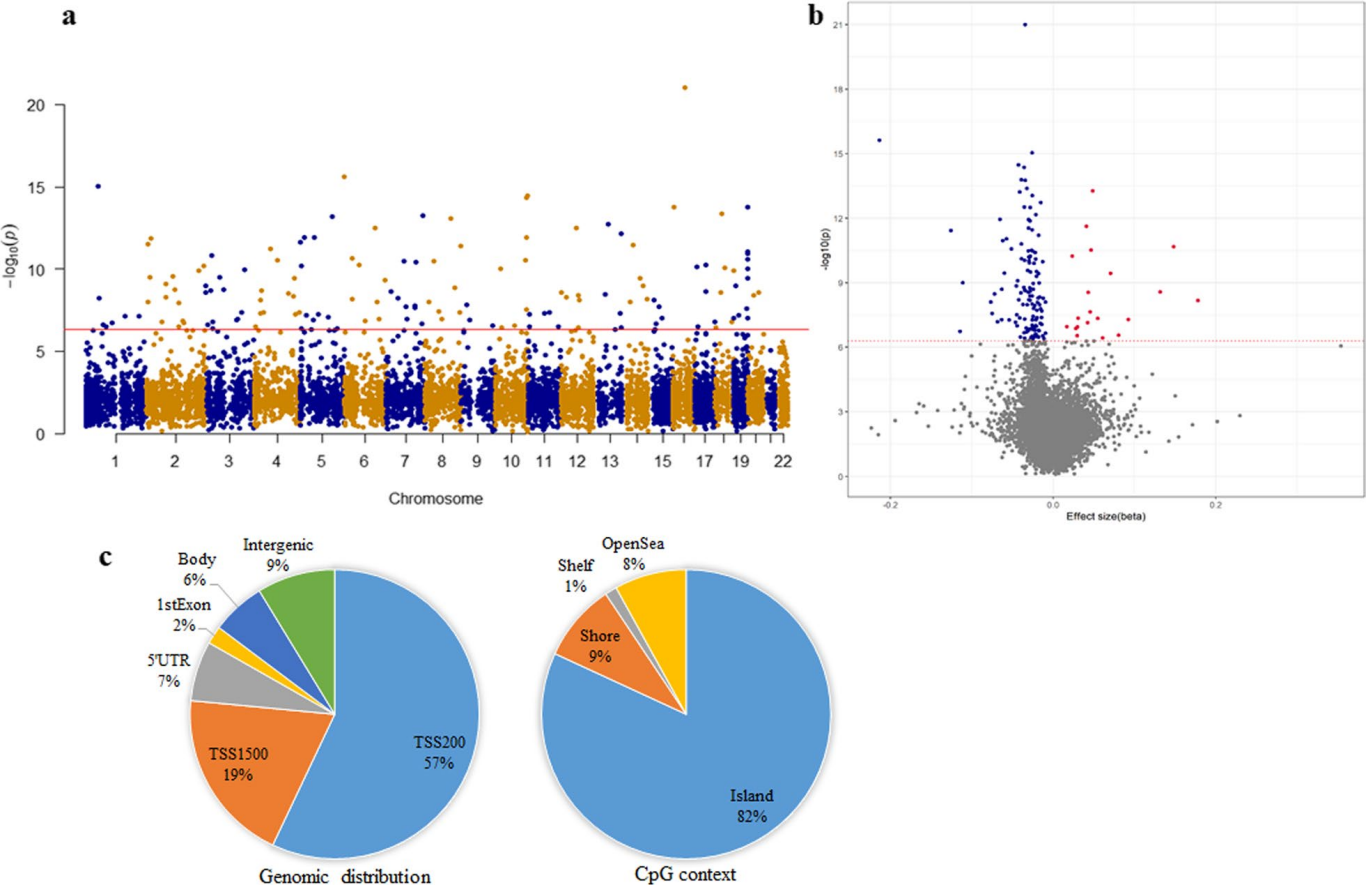
Manhattan (**a**) and volcano (**b**) plots displaying the differentially methylated regions (DMRs) associated with ischemic stroke in the discovery sample of 80 Chinese adults (40 cases *vs*. 40 matched controls). In the Manhattan plot, X-axis indicates the chromosome position; Y-axis indicates the –log_10_ *P*-values. The red line denotes the significance threshold at q-value < 0.05. In the volcano plot, blue and red dots highlight significantly hypomethylated and hypermethylated regions, respectively, in cases with respect to controls. Genomic distribution and CpG context (**c**) of the identified DMRs indicated that these DMRs are mainly located in the first exon, promoter, and CpG Islands

Region-based analysis identified 149 DMRs (annotated to 168 unique genes) significantly associated with IS at q < 0.05 (Fig. 5a, Additional file 1: Table S1). Of these, 129 DMRs were hypomethylated and 20 DMRs were hypermethylated in relation to IS (Fig. 5b). The identified DMRs are mainly located in the first exon and promoter regions. With respect to CpG context, the identified DMRs are largely located within CpG Islands (Fig. 5c).

### Functional annotation of the differentially methylated genes associated with IS

The 7 DMPs and 149 DMRs identified in the discovery sample were annotated to 170 unique genes. These genes were significantly enriched in 351 GO biological processes including neuron differentiation, neurogenesis, and cell–cell signaling (Additional file 1: Table S2), 37 GO cellular components including neuron and cell junction (Additional file 1: Table S3), and 12 GO molecular functions including DNA binding transcription factor activity (Additional file 1: Table S4). The same gene set was also enriched in several GTEx tissues, including brain, thyroid, nerves, and blood vessels (Additional file 1: Table S5). Finally, the identified differentially methylated genes were significantly enriched in GWAS risk genes associated with stroke-related phenotypes such as chronotype, obesity-related traits, coronary artery calcification, and blood pressure (Additional file 1: Table S6). These findings provide further support for the potential functional role of the identified genes in IS.

## Discussion

In this large-scale EWAS, we employed a multi-stage approach to identify peripheral blood DNA methylation patterns associated with IS in Chinese population. Specifically, we identified DNA methylation at 7 CpGs (annotated to 7 unique genes) significantly associated with IS. Of these, 6 CpGs (cg15137954 in *TRIM6*, cg07913294 in *AGBL4*, cg11533098 in *FAM84A*, cg24891539 in *SOX17*, cg16800165 in *FLRT2*, and cg15949239 in *TLN2*) were confirmed in an independent Chinese population, and one CpG (cg15137954, located in the *TRIM6* gene) was further verified in an independent cohort of European ancestry. Functional analysis in cellular models via the CRISPR/dCas9-Dnmt3a system revealed that DNA methylation at three genes (*TRIM6*, *FLRT2,* and *TLN2*) harboring the aberrantly methylated probes could regulate the expression of three endothelial cell adhesion markers (i.e., *ICAM1*, *VCAM1*, and *SELE*) and thus be functionally implicated in endothelial cell activation and leukocyte transendothelial migration [28], one of the mechanisms underlying atherosclerosis [29, 30] and stroke [31].

Of the differentially methylated genes identified, the observed association of DNA hypomethylation of the tripartite motif containing 6 (*TRIM6)* gene with IS is especially interesting. This gene belongs to the *TRIM* gene family, which has been associated with innate immunity, a mechanism known to be implicated in IS and atherosclerosis [32]. In this study, we found that experimentally induced increased DNA methylation at the *TRIM6* locus resulted in decreased expression of the *TRIM6* gene as well as reduced expression of genes encoding two endothelial cell adhesion markers (*VCAM1* and *SELE*). These results point to an increased expression of *TRIM6*, *VCAM1*, and *SELE* in IS cases. The *TRIM6* gene was confirmed in both internal and external replications and further verified in the cellular models. Previous research in human cells has shown that several TRIM family genes (e.g., *TRIM5, TRIM34*) were upregulated in response to interferon gamma (IFN-γ) and lipopolysaccharide (LPS), while *TRIM5* protein was involved in the containment of viral spread in rhesus monkeys [33, 34]. Of note, acute viral infections have been suggested as potential triggers for IS due to destabilization and rupture of atherosclerotic plaques in humans [35, 36]. In addition, genetic polymorphisms in the *TRIM5*/*TRIM6* locus have previously been associated with coronary artery disease [37]. Moreover, *TRIM6* has been reported to aggravate myocardial ischemia/reperfusion injury in mice [38]. A genetic polymorphism at another TRIM family member (*TRIM36*) has recently been associated with cardioembolic stroke, a subtype of ischemic stroke, in a genome-wide association analysis [39]. Together, these findings support a causal role of altered *TRIM6* gene methylation in the pathogenesis of IS.

The Talin 2 (*TLN2*) gene encodes a protein related to talin 1, a cytoskeletal protein that is involved in the assembly of actin filaments and the spreading and migration of various cell types, and may play an important role in cell adhesion [40]. Differential expression of the *TLN2* gene was previously reported to modulate carotid intima media thickness in European individuals [41]. Moreover, downregulation of the *TLN2* gene has been associated with atherosclerosis in human arterial plaque tissue samples [42]. In the present study, we found that stroke patients were characterized by *TLN2* hypermethylation, while in vitro modulation of DNA methylation at the *TLN2* locus via CRISPR/dCas9-Dnmt3a system revealed a positive association between *TLN2* methylation and *TLN2* expression. Moreover, *TLN2* methylation was negatively correlated with the expression of genes encoding endothelial cell adhesion markers. The *FLRT2* (fibronectin leucine-rich transmembrane protein 2) gene is expressed in pancreas, skeletal muscle, brain, and heart [43]. This gene was previously found to be involved in the regulation of early embryonic vascular and neural development [43], and a genetic polymorphism of the *FLRT2* gene has been associated with IS in African-Americans [44]. In this study, we found that stroke patients exhibited lower methylation level of the *FLRT2* gene compared to controls. Additionally, increased *FLRT2* methylation was associated with decreased *FLRT2* expression and increased expression of genes encoding endothelial cell adhesion markers. Together, these findings suggest that the observed changes in DNA methylation in *TLN2* and *FLRT2* genes in IS patients are associated with decreased gene expression levels of cell adhesion markers. These observations support the role of DNA methylation in responding to ischemic injury and highlight its potential role in stroke prognosis rather than its pathogenesis.

With regard to DMR findings, out of 149 DMRs identified, 2 DMRs were located in genes previously associated with stroke in a multi-ancestry GWAS: *FBN2* and *PDE3A* genes [39]. Interestingly, the SNP rs55670004 previously associated with IS has been identified as a methylation quantitative trait loci (mQTL) for CpG sites cg02271895 and cg17564775, both of which are included in the *FBN2* DMR (chr5:127,872,329–127,875,163) associated with IS in the current study. No mQTLs were found for *PDE3A* gene. These results highlight that at least some of the identified DMRs are driven by the underlying genetic background of assessed participants suggesting that DNA methylation at these regions might play a causal role in stroke etiology. Additionally, 17 out of 168 unique genes located in close proximity to identified DMRs were already reported by a previous EWAS of IS in a Chinese population [11]. Of note, one of these 17 overlapping genes was *FBN2*, further highlighting its role in IS etiology.

In line with previous research [12], we found that the majority of the genes identified in our study, both in the DMP and DMR approaches, are hypomethylated with respect to stroke status (i.e., DNA methylation was lower in cases than in controls). The identified genes are significantly enriched in biological pathways involved in neurogenesis, neuron differentiation, and neuronal development, as well as nervous cellular components, such as neurons and synapses. These findings suggest that peripheral blood DNA methylation may serve as a proxy for DNA methylation in the brain, the target organ of stroke. Further studies in postmortem brain tissue will reveal whether the observed DNA methylation changes in blood of IS survivors are also present in brain tissue of deceased participants that underwent IS. Other pathways highlighted included cell adhesions, cell junctions, and extracellular matrix, all of which are known to be involved in leukocyte transendothelial migration and atherogenesis, a major contributor to IS [45]. Several pathways identified in the present study were also reported in a previous EWAS of IS in a Chinese population, including synapse assembly, synapse organization, and homophilic cell adhesion [11]. GWAS trait enrichment further revealed that genes associated with chronotype and sleep disturbances might be differentially methylated in IS participants. Chronotype, also known as diurnal preference, refers to activity-rest preference in a given 24-h period [46]. Of note, several sleep disturbances have previously been identified as a risk factor for stroke onset [47], while sleep duration has been linked with subclinical atherosclerosis [48]. Disruption of circadian rhythms in shift workers and in experimental models in mice and sheep increased reperfusion injury after myocardial infarction providing experimental evidence of the causal link between poor sleep patterns and worse prognosis after ischemic events [49]. Finally, the observation that several risk factors for stroke (i.e., obesity, body mass index, blood pressure, and smoking status, as summarized in Additional file 1: Table S6) were enriched in gene pathway analysis suggests that DNA methylation might mediate the deleterious effects of such risk factors, thus increasing IS risk.

Several limitations of our study merit discussion. First, DNA methylation was measured after the onset of stroke. Due to the dynamic nature of epigenetic mechanisms, identified differentially methylated genes could be involved in either stroke pathogenesis, ischemic injury, or neuroprotection following IS. Further prospective longitudinal studies are warranted to test causality of observed DNA methylation changes. However, findings from our CRISPR/dCas9-Dnmt3a cell model suggest that the observed DNA methylation changes have antagonistic effects suggesting that we identified a mixture of stroke inducing (e.g., *TRIM6* gene) as well as neuroprotective (e.g., *TLN2* and *FLRT2* genes) epigenetic biomarkers. Second, given the tissue and cell type specificity of DNA methylation, blood DNA methylation may not reflect the changes in human brain. Nonetheless, previous research has shown that blood DNA methylation may partially reflect changes in the targeted tissue and thus can serve as proxy non-invasive markers [50]. Third, participants were not stratified by stroke etiology (i.e., large artery, small vessel, cardioembolic strokes). Fourth, it is well established that nutrition could not only affect DNA methylation but also contribute to the risk of stroke. Unfortunately, data on diet were not available in the cohorts involved in our study. We cannot prevent the confounding effects caused by nutrition, as well as other unmeasured confounding, on the association between DNA methylation and stroke in an observational study. Fifth, the sample size of the discovery cohort is relatively smaller than prior EWAS studies. However, we conducted an independent replication study with a larger sample size to exclude the probable false-positive results. All DMPs identified in the discovery cohort were successfully replicated, indicating that the small sample size of the discovery cohort may not affect our findings a lot. Finally, although we included an external validation, only one DMP was nominally associated with IS (same direction) in the European population. The lack of replication could be attributed to differences in genetic makeup, clinical diagnosis, environmental exposures, and recruitment procedures between the study populations. For example, blood collection following stroke was performed within 12 h in the European cohort and within 48 h in the Chinese cohort. Given the known epidemiological differences in stroke incidence among different countries, with stroke being the leading cause of mortality in China, it is possible that some of the reported genes herein are specific to the Chinese population. The robustness of our multi-stage study design, including initial discovery, technical verification by TBS, internal and external validation in human populations, as well as functional validation in vascular endothelial cell models supports the strength of the reported findings.

In summary, we identified novel genes associated with IS in Chinese population. These findings enhance the understanding of DNA methylation involvement in both stroke pathogenesis and response to ischemic injury and highlight the importance of functional validation in epigenome-wide association studies in future research.

## Methods

### Participants

The study design and selection of participants are illustrated in Fig. 1. In the discovery stage, 40 IS cases and 40 controls were randomly selected from the China Antihypertensive Trial in Acute Ischemic Stroke (CATIS, 2009–2016) and the Prevention of Metabolic syndrome and Multi-metabolic disorders Study (PMMS, 2000–2004), to identify potential epigenetic markers of IS. From the remaining CATIS and PMMS participants, 1000 cases and 1000 age- and sex-matched controls were randomly selected as an internal replication sample to further confirm epigenetic markers of IS; after QC, 853 cases and 918 controls remained for downstream analysis. Additionally, an external cohort of 390 Spanish participants was used for further validation of the DMPs identified. To test the causal effect on gene expression of DNA methylation at loci identified in the aforementioned stages, human umbilical vein endothelial cell (HUVEC) models were epigenetically modified via CRISPR/dCas9-Dnmt3a.

*Chinese cohorts* (discovery and internal validation) included adults from the China Antihypertensive Trial in Acute Ischemic Stroke (CATIS, 2009–2016) and the Prevention of Metabolic syndrome and Multi-metabolic disorders Study (PMMS, 2000–2004). Detailed study design and methods for both cohorts have been described elsewhere [14, 15]. Briefly, CATIS is a multicenter randomized clinical trial (NCT01840072) designed to test whether moderate lowering of blood pressure in the acute phase after the onset of IS can reduce mortality and major disability at 14 days or hospital discharge. A total of 4,071 patients who had first-ever IS within 48 h of symptom onset and had an elevated systolic blood pressure between 140 mmHg and less than 220 mmHg were recruited to the CATIS. PMMS is a community-based cohort study including 5,888 Chinese adults free of overt cardiovascular disease (CVD) at the time of enrollment. All participants in the CATIS and PMMS reside in the Changshu city, Jiangsu Province, China.

The *European cohort* (external replication) included Spanish individuals of European ancestry from the BasicMar Register and the Girona Heart Registry study (REGICOR). Detailed information for these cohorts has been described elsewhere [16, 17]. Briefly, BasicMar is a hospital-based registry of stroke patients admitted to the Hospital del Mar in Barcelona, Spain [16]. REGICOR is a population-based registry recruiting participants residing in Girona, Spain [17]. REGICOR participants with no previous history of IS and no previous history of acute myocardial infarction were included as controls in the current analysis.

### Study approval

The study protocols of CATIS were approved by the Institutional Review Boards at Soochow University in China and Tulane University in the USA. The study protocols of PMMS were approved by the Soochow University Ethics Committee. Written informed consent was obtained from all study participants. The study protocols of BasicMar and REGICOR were approved by the ethics committees of all participating hospitals, and all participants provided written informed consent to participate.

### Clinical data and blood sample collection

For participants in the Chinese cohorts, demographic and lifestyle information and disease history were collected using standard questionnaires, as previously described [18]. For CATIS participants, blood samples were collected within 48 h of stroke onset and then stored at -80 °C for biochemical and DNA methylation analysis. Fasting plasma glucose and blood lipids including total cholesterol, triglycerides, high-density lipoprotein cholesterol (HDL-C), and low-density lipoprotein cholesterol (LDL-C) were measured by standard laboratory methods [19]. IS was ascertained by computed tomography (CT) or magnetic resonance imaging (MRI) of the brain.

For participants in the BasicMar and REGICOR cohorts, information for disease history including vascular risk factors was obtained from personal interviews to the patients, relatives, or caregivers, or abstracted from medical records. IS diagnosis was confirmed by CT or MRI. Blood samples were collected at hospital arrival, in the acute phase of the stroke (within 12 h of symptoms onset).

### Epigenome-wide association study and confirmation by targeted bisulfite sequencing

#### Discovery stage

Forty cases and 40 age- and sex-matched controls were randomly selected from the CATIS and PMMS, respectively, for epigenome-wide association analysis. Briefly, genomic DNA was extracted from whole peripheral blood using the EZgene™ Blood DNA Kit (Biomega, Inc.), followed by bisulfite treatment using the EZ DNA Methylation-Gold Kit (Zymo Research, Orange, CA, USA). DNA methylation was quantified by the Infinium HumanMethylationEPIC BeadChip array (Illumina, Inc., San Diego, CA). Raw DNA methylation data were processed using the R Bioconductor package “minfi” (v1.30.0) [20]. After quality control and exclusion of probes on the sex chromosomes, a total of 815,017 probes were used in the downstream analysis. Blood cell proportions (CD8^+^ T cells, CD4^+^ T cells, natural killer cells, B cells, monocytes, and granulocytes) were estimated using the Houseman’s estimation method [21].

#### Fine mapping and internal replication

In an independent sample comprising 1000 cases from the CATIS and 1000 age- and sex-matched controls from the PMMS, targeted bisulfite sequencing (TBS) [22] was performed to confirm and discover more differentially methylated probes (DMPs) associated with IS. Briefly, primers were designed to detect the maximum number of additional CpG loci in the vicinity of identified DMPs. TBS target gene sequences and primers can be found in Additional file 1: Table S7. Genomic DNA was bisulfite-treated using the EZ DNA Methylation-Gold Kit (Zymo Research, Inc., CA, USA) according to the manufacturer’s protocol. Samples were then amplified, barcoded, and sequenced by Illumina Hiseq 2000 (Illumina, Inc., CA, USA) using the paired-end sequencing protocol according to manufacturer’s guidelines. Methylation level at each CpG locus was calculated as the percentage of the methylated alleles over the sum of methylated and unmethylated alleles. For quality control, the samples with bisulfite conversion rate < 98% and the cytosine sites with average coverage less than 20 × were filtered out. After quality control, 853 cases and 918 controls remained for downstream analysis; all CpG sites passed quality control. Demographic and clinical characteristics of included (n = 1771) vs. excluded participants (n = 229) can be found in Additional file 1: Table S8.

#### External replication

DNA methylation in the BasicMar and REGICOR was assessed by Infinium MethylationEPIC Beadchip arrays (Illumina Netherlands, Eindhoven, Netherlands), as previously reported [9]. A total of 390 individuals, including 207 cases from BasicMar and 183 controls from REGICOR, were included in the external replication analysis.

### Functional validation of DMPs in cellular models

To examine the potential functional role of the identified CpGs in vascular endothelial cell activation and inflammation, we employed the CRISPR/dCas9-Dnmt3a system to develop site-specific methylation models in a culture of human umbilical vein endothelial cells (HUVEC).

#### Construction of CRISPR/dCas9-Dnmt3a plasmids

The dCas9-5xGCN4 fusion protein expression vector (ZP509, pEFS-dCas9-5xGCN4-T2A-EGFP) was generated by fusing cDNA encoding the catalytically inactive nuclease codon-optimized S. pyogenes Cas9 (dCas9) to quintuple tandem short peptide GCN4 in an expression vector using the EFS promoter. The 5xGCN4 fragment was synthesized by Genewiz and cloned into pEFS-dCas9-EGFP (Pregen plasmid: ZP508) with BamHI sites by Gibson Assembly to package lentiviruses (Pregen plasmid: ZP509). The aa sequence of GCN4 used was EELLSKNYHLENEVARLKK. The sequences of the linker between each GCN4 peptide unit were GSGSGGSGSGGSGSGSGGSGSGGSGSG. All the fusion proteins were expressed under the control of the EFS promoter. The DNMT3L CD-DNMT3ACD fragment was amplified from Addgene plasmid: 112,210. The scFv GCN4 fragment was amplified from Addgene plasmid 60,904. The two PCR fragments were cloned into pEFS-mCherry (Pregen plasmid: ZP510) with AgeI and RsrII sites by Gibson Assembly to package lentiviruses (Pregen plasmid: ZP512). Once expressed, the scFv GCN4 minimal antibody fused to DNMT3A binds to the GCN4 tandem repeats fused to dCas9, thus allowing colocalization of the inactive nuclease dCas9 with the DNA methyltransferase in transfected cells.

#### Construction of gRNAs

The single-guide RNA (sgRNA) expression vectors for *TRIM6*, *SOX17*, *TLN2*, *SOX1*, *AGBL4*, *FLRT2*, *FAM84A*, and negative control (NC) were cloned by inserting the target sequences into ZP512 plasmid. Cloning was performed by linearization of an PacI site and Gibson Assembly-mediated incorporation of the gRNA insert fragment. All constructs were sequenced before transfection. The sequences for primers and sgRNA used for CRISPR/dCas9-Dnmt3a can be found in Additional file 1: Table S9.

#### Production of lentiviral particles

HEK293T immortalized cells were incubated in DMEM with 10% FBS and 1% P/S. All cells were grown at 5% CO2 and 37 °C incubation. The HEK293T cells were transfected with either the pEFS-dCas9-5xGCN4-EGFP plasmid or the pU6-sgRNA-EFS-scFvGCN4-DNMT3L-DNMT3a-mCherry plasmid with different sgRNAs sites using polyethyIenimine (PEI polysciences #23966). Lentiviral particles were collected at 48 h and 72 h after transfection. Viral particles were then concentrated by centrifugation at 25,000 g for 2 h at 4 °C and resuspended in PBS medium.

#### Lentivirus transfection and flow sorting

HUVEC cells (LONZA #CC-2519) were grown in EBM™ Basal medium (LONZA, #CC-3121) supplemented with 10% fetal bovine serum (FBS) and 1% penicillin/streptomycin (P/S). HUVEC cells were co-infected with various lentiviral mixtures, transfected with both plasmids with different gRNAs sites for 72 h. EGFP and mCherry double-positive cells were obtained by flow sorting and were cultured and expanded for 5 generations. After expansion, the HUVEC cells were used in downstream functional analysis.

#### Pyrosequencing

We then verified the presence of site-specific methylation at our region of interest by pyrosequencing to check the validity of the cellular model. Briefly, genomic DNA of the isolated cells was extracted using a DNA Mini Kit (QIAGEN 51304) and then bisulfite converted using an EpiTect Bisulfite Kit (Qiagen 59,104) according to the manufacturer’s instructions. Bisulfite PCR primers and their target gene sequences are listed in Additional file 1: Table S10. Twenty-five microliter 2xTaq DNA polymerase premix, 1 μl for each forward and reverse primer, 21 μl double-distilled water (ddH2O), and 2 μl bisulfite-treated DNA were mixed and run according to the following PCR program. First, samples were heat activated at 95 °C for 3 min; for the second step, they were kept at 94 °C for 30 s, then at 56 °C for 30 s, and then at 72 °C for 1 min. Then the process was repeated from the second step for 40 cycles and finally extended at 72 °C for 7 min. Bisulfite PCR products were immobilized on streptavidin beads, and single-stranded DNA was prepared, sequenced, and finally analyzed on the PyroMark Q96 ID system (Qiagen). For quality control, each experiment included non-CpG cytosines as an internal control to verify completion of sodium bisulfite DNA conversion.

#### Quantitative real-time PCR (qRT-PCR)

Finally, we quantified the expression levels of candidate genes harboring the putative DMPs by quantitative real-time polymerase chain reaction (qRT-PCR). Additionally, the expression levels of genes encoding endothelial cell adhesion markers, including ICAM1 (intercellular adhesion molecule 1), VCAM1 (vascular cell adhesion molecule 1), and SELE (selectin E), were also measured by qRT-PCR in the human umbilical vein endothelial cells (HUVECs). The *Actin* gene of human species was used as a control. Specific primers were designed based on cDNA sequences. Primer sequences for qRT-PCR experiments can be found in Additional file 1: Table S11. The qRT-PCR was performed on a Bio-Rad S1000 with Bestar SYBR Green RT-PCR Master Mix (TOYOBO). PCR conditions consisted of denaturing at 95 °C for 1 min and 40 cycles of denaturing at 95 °C for 15 s followed by annealing and extension at 60 °C for 30 s. Relative gene expression was calculated using the Livak and Schmittgen 2 − ΔΔCt method [23], normalized with the reference gene Actin. PCR amplifications were performed in triplicate for each sample.

### Statistical analysis

#### Single-probe analysis

To identify DMPs associated with IS, we constructed multivariate linear regression models via the R package limma [24]. In the model, DNA methylation level at each CpG was the dependent variable and status of IS was the independent variable. The model adjusted for age, sex, BMI, smoking, alcohol consumption, hypertension, LDL-C, HDL-C, fasting plasma glucose, estimated cell counts, and surrogate variables derived from surrogate variable analysis (SVA) [25]. Multiple testing was controlled by false discovery rate (FDR), and FDR-adjusted *P*-value (i.e., q-value) < 0.05 was considered statistically significant.

#### Region-based analysis

Differentially methylated regions (DMRs) were identified using the program DMRcate [26]. The program identifies genomic regions harboring putative disease-associated CpGs and takes into consideration the correlations between adjacent probes. Here we defined a DMR as a region containing at least 2 correlated probes (adjacent peak probes [*P* < 0.05] spaced less than 1,000 bp). A DMR with *P*-value < 5.0 × 10^–7^ was considered statistically significant.

#### Enrichment and pathway analysis

To functionally annotate the identified differentially methylated genes, we conducted functional enrichment analysis using the computational program FUMA [27]. We defined differentially methylated genes as unique genes containing either DMPs or DMRs identified above. P-value < 0.05 was used to determine statistical significance for the annotated biological pathways.

## Supplementary Information

**Additional file 1**. The supplemental tables showing the additional results.

## Data Availability

The datasets used during the current study are available from the corresponding author on reasonable request.
